# Can the combination of proanthocyanidin and vitamin E or palm oil effectively protect enamel against *in vitro* erosive and abrasive challenges?

**DOI:** 10.1590/1678-7757-2024-0100

**Published:** 2024-07-22

**Authors:** Daiana da Silva MARTINS, Ana Paula BOTEON, Amanda Moura FERREIRA, Ana Luiza Bogaz DEBORTOLLI, Isabella Claro GRIZZO, Franciny Querobim IONTA, Thiago Saads CARVALHO, Marilia Afonso Rabelo BUZALAF, Daniela RIOS, Heitor Marques HONÓRIO

**Affiliations:** 1 Universidade de São Paulo Faculdade de Odontologia de Bauru Departamento de Odontopediatria, Ortodontia e Saúde Coletiva Bauru SP Brasil Universidade de São Paulo, Faculdade de Odontologia de Bauru, Departamento de Odontopediatria, Ortodontia e Saúde Coletiva, Bauru, SP, Brasil.; 2 Universidade de Marília Departamento de Odontopediatria da UNIMAR Marília SP Brasil Universidade de Marília, Departamento de Odontopediatria da UNIMAR, Marília, SP, Brasil.; 3 University of Bern Department of Preventive, Restorative and Pediatric Dentistry Bern Switzerland University of Bern, Department of Preventive, Restorative and Pediatric Dentistry, Bern, Switzerland.; 4 Universidade de São Paulo Faculdade de Odontologia de Bauru Departamento de Departamento de Ciências Biológicas Bauru SP Brasil Universidade de São Paulo, Faculdade de Odontologia de Bauru, Departamento de Departamento de Ciências Biológicas, Bauru, SP, Brasil.

**Keywords:** Vitamin E, Polyphenols, Palm oil, Dental pellicle, Tooth erosion, Tooth abrasion, Erosive tooth wear

## Abstract

**Objectives:**

This study aimed to assess the effect of proanthocyanidin, palm oil and vitamin E against erosive and erosive+abrasive challenges *in vitro* after enamel pellicle formation *in situ*.

**Methodology:**

Bovine enamel blocks (n=84) were obtained and divided into the following treatment groups: negative control (NC) - deionized water; positive control (PC) - SnCl2/NaF/AmF-containing solution; palm oil (PO); 2% proanthocyanidin (P2); vitamin E (VitE); 2% proanthocyanidin+palm oil (P2PO); and 2% proanthocyanidin+vitamin E (P2VitE). For 5 days, one half of the sample from each group was subjected to erosion and the other half was subjected to erosion+abrasion. The acquired enamel pellicle (AEP) was pre-formed *in situ* for 30 minutes. The specimens were then treated *in vitro* with solutions (500 µl, 30s for each group). Subsequently, the blocks were left in the oral cavity for another hour to obtain the modified AEP. The blocks were immersed in 0.5% citric acid (pH=2.5) for 90s, 4×/day. AEP formation and treatment were carried out before the first and third erosive challenges, and after these challenges, abrasive cycles (15s) were performed on half of the samples. Enamel wear was quantified by profilometry and data were analyzed by two-way ANOVA and Tukey’s test (p<0.05).

**Results:**

All groups showed higher wear when exposed to erosion+abrasion than when exposed to erosion alone (p=0.0001). PO, P2VitE, P2, and P2PO showed enamel wear similar to the PC group, but only PC, PO and P2VitE differed from the NC group. The other groups behaved similarly to NC.

**Conclusion:**

It was concluded that the combination of proanthocyanidin and vitamin E was effective in reducing wear in the face of *in vitro* erosive and erosive+abrasive challenges.

## Introduction

During the functions of the stomatognathic system, such as chewing, teeth face erosive challenges due to exposure to acidic foods and beverages, as well as mechanical challenges.^1-3^ Conversely, the body uses various mechanisms, including the production of saliva, to protect teeth from these challenges^4-8^ As a result, in a healthy state, there is gradual and minimal wear of the dental structure over time. This wear does not compromise the aesthetics, function, or quality of life of individuals.^9^ Moreover, situations of imbalance can occur, with acidic and mechanical challenges exceeding the protective capacity, leading to adverse effects on dental health.^10^ In such scenarios, the optimal therapeutic approach involves mitigating both mechanical and acidic challenges via dietary management and habit control associated with attrition and abrasion. This invariably requires the affected individuals to change their behavior.^9^ However, an alternative branch of recent research explores the enhancement of oral defense mechanisms, specifically by fortifying the acquired salivary pellicle.^11-15^

Proanthocyanidin is a polyphenolic compound^13,16-18^ derived from a variety of sources, including fruit, vegetables, peel, and seeds, mainly grape seeds.^19^ Proanthocyanidin is considered a natural cross-linking agent due to its ability to precipitate proline-rich proteins (PRPs), mainly by hydrogen bonds,^20-23^ and it can form insoluble complexes with carbohydrates and proteins.^24^ The interaction between proanthocyanidin and proteins enhances the protective effect of the acquired enamel pellicle (AEP)^19^ against dental erosion, as evidenced by previous studies.^13,25^

On the other hand, lipids account for 25% of the composition of AEP^26^ and may play a significant role in its protective function, with an enhanced ability to provide protection against acidic challenges.^7,11,12,14,27,28^ Previous research has demonstrated that palm oil has the ability to protect against enamel erosion^11,12,14^ and erosion associated with abrasion.^12^ This effect has been attributed to its composition rich in tocotrienols,^29^ which diffuse via the AEP, thereby retarding its degradation.^11,12^ However, despite its low cost and widespread availability, palm oil is associated with certain unfavorable properties, such as an unpleasant taste and the potential to stain teeth, which may limit its practicality in oral hygiene products. As a result, recent investigations have aimed to identify the specific component responsible for this protective effect, and vitamin E has been identified as a potential candidate.^14,15^

Given the demonstrated protective potential of proanthocyanidin, palm oil, and vitamin E and the low cytotoxicity of these natural products,^18,30,31^ there is considerable interest in investigating whether their combined use can enhance protection against enamel erosion and abrasion-related erosion. Therefore, this study aimed to evaluate the impact of proanthocyanidin and palm oil or vitamin E, individually and in combination, on enamel erosion and erosion combined with abrasion in an *in vitro* setting, using an *in situ* enamel pellicle acquisition model. The null hypotheses were as follows: (H1) Proanthocyanidin and palm oil or vitamin E, alone or in combination, do not protect enamel against erosion; (H2) Proanthocyanidin and palm oil or vitamin E, alone or in combination, do not protect enamel against erosion combined with abrasion.

## Methodology

### Experimental design

This study adhered to the principles of the Declaration of Helsinki.^32^ The study protocol was approved by the local research ethics committee (Protocol 57816322.7.0000.5417). All subjects signed an informed consent form prior to confirmation of their eligibility to participate in the study.

**Figure 1:**
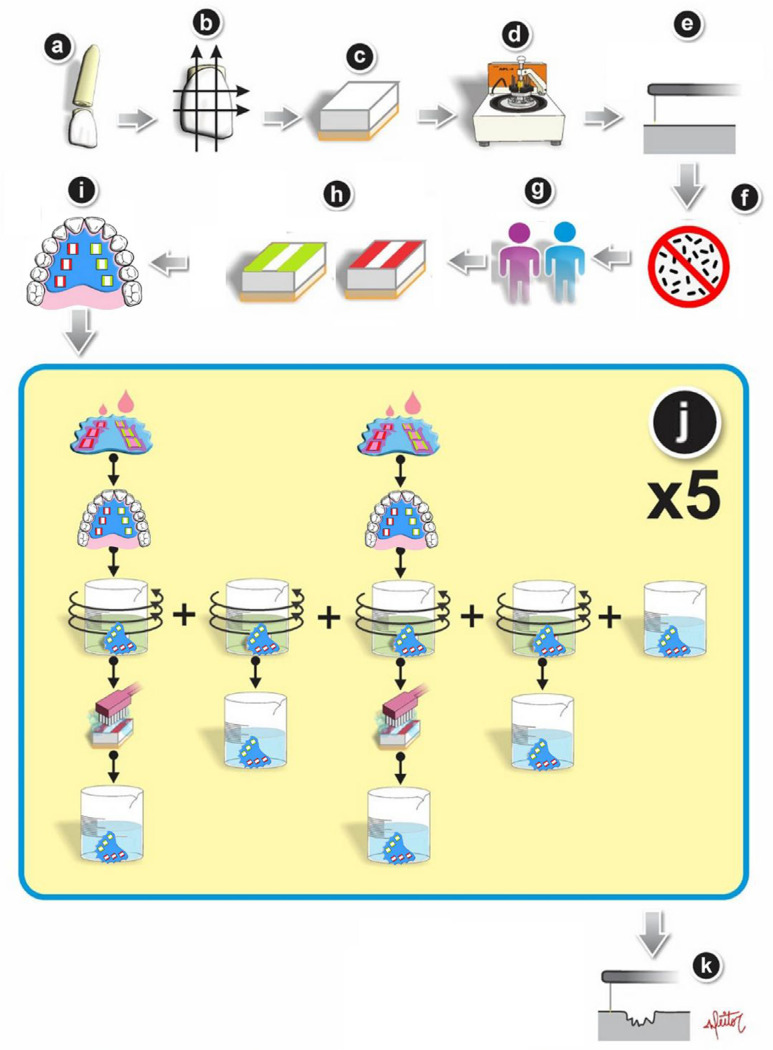
Illustration of the experimental design of the study a. Separation of the bovine teeth into crown and root sections; b. Marking the area on the crown to be cut; c. Cutting enamel blocks measuring 4x4 mm2; d. Polishing the specimens; e. Initial profilometric analysis; f. Sterilizing the specimens; g. Recruiting and fitting volunteers for the study; h. Applying nail polish to create reference areas (green for erosion, red for erosion+abrasion); i. Using the intraoral appliance for 30 minutes to establish the initial acquired pellicle; j. Treating the specimens with solutions (30 seconds), using the intraoral appliance for 1 hour to develop the modified pellicle, conducting four erosive cycles (90 seconds each), and performing abrasive cycles (15 seconds, 45 back-and-forth movements, with a weight of 150g); k. Conducting the final profilometric analysis.

Figure 1 shows the experimental design. The factors studied were treatment at seven levels (deionized water; SnCl2/NaF/AmF-containing solution; palm oil; 2% proanthocyanidin; vitamin E; 2% proanthocyanidin+palm oil; 2% proanthocyanidin+vitamin E) and wear condition at two levels (erosion; erosion and abrasion). The sample unit was the enamel block, and the sample size was determined considering seven study groups with a minimum detectable difference of 1.5 µm of enamel loss, a standard deviation of 0.87 (values obtained in a pilot study), an α error of 5%, and a β error of 20%. This calculation resulted in 11 blocks per group; however, to improve specimen distribution, 12 specimens were used per treatment group, for a total of 84 enamel blocks. Enamel pellicle acquisition was performed *in situ* using an intraoral palatal appliance by two volunteers for 30 minutes twice a day (for 5 days), with six enamel blocks in each appliance (three for erosion and three for erosion+abrasion). The blocks were treated *in vitro* according to the following groups: negative control (NC) - deionized water; positive control (PC) - SnCl2/NaF/AmF-containing solution (Elmex^®^ Erosion Protection Dental Rinse/EP - CP GABA GmbH; Hamburg, Germany); palm oil (PO) Kidendê - Dendê Light Indústria de Produtos Alimentícios Ltda; Valença, BA, Brazil); 2% proanthocyanidin (P2) (95% dry extract *Vitis vinifera* - Florien Fitoativos; Piracicaba, SP, Brazil); vitamin E (VitE) (97.8% oily tocopherol acetate - Lepuge Insumos Farmacêuticos Eireli; São Bernardo do Campo, SP, Brazil); 2% proanthocyanidin+palm oil (P2PO); and 2% proanthocyanidin+vitamin E (P2VitE). All solutions were applied using a dropper (five drops) to ensure complete coverage of the enamel surface, with each application lasting 30 seconds, which is the recommended application time for Elmex^®^ Erosion Protection. After treatment, the appliance was tilted at 90 degrees to facilitate the removal of excess solution. Subsequently, the appliance was reinserted into the oral cavity for an additional hour to allow for the formation of the modified AEP.

All specimens were subjected to four erosive cycles per day for five consecutive days, with immersion in 30 ml citric acid (0.5%, pH 2.5) for 90 seconds during each cycle. Abrasive cycles were performed on half of the sample, with simulated brushing following the first and third erosive cycles. Enamel pellicle formation and treatment were conducted only after the first and third cycles. Enamel wear was then analyzed using contact profilometry.

### Preparation of enamel blocks

Enamel blocks (4x4x3mm^3^, n=84) were obtained from the labial surfaces of bovine incisor crowns. The blocks were sectioned using two diamond discs (Extec Cor.; Enfield, Connecticut, United States) separated by a 4 mm-thick spacer on an IsoMet^®^ low-speed saw-cutting machine (Buehler Ltd.; Lake Bluff, Illinois, United States). After sectioning, the enamel block surfaces were further smoothed with water-cooled silicon carbide discs (320, 600, and 1200 grade papers; Buehler Ltd.; Lake Bluff, Illinois, United States) and then polished with felt paper and diamond spray (1 µm; Buehler Ltd.; Lake Bluff, Illinois, United States). Specimens with cracks, fractures, or dentin exposure were excluded. Each block was assigned a unique identification number ranging from 1 to 84 and then randomized among treatment groups (NC, PC, PO, P2, VitE, P2PO, and P2VitE), condition types (erosion or erosion+abrasion), and two different volunteers. The blocks were then sterilized by exposure to ethylene oxide gas.

### Baseline profilometric analysis

The enamel block surfaces were marked with a No. 11 scalpel blade (Embramac, Itapira, SP, Brazil) to delineate reference areas of 1.0 mm (at the periphery) and a test area of 2.0 mm (at the center). The initial profile of the enamel blocks was assessed using a profilometer (Marh, MarSurf GD 25, Göttingen, Germany) and contouring software (MarSurf XCR 20) with detection limits of 0.5 µm.^33^ To ensure positional standardization, the blocks were secured in a special holder and their positions were recorded to facilitate precise repositioning after the *in vitro* phase. Five measurements were taken for each block at predefined intervals of 2.25, 2.0, 1.75, 1.5, and 1.25 µm, relative to the position of the block along the y-axis. After the initial profilometry analysis, to preserve the enamel in the reference areas, these regions (at the periphery of the block) were coated with nail polish (Maybelline Colorama, Cosbra Cosmetics Ltda, São Paulo, SP, Brazil), using green for blocks subjected only to erosion and red for blocks subjected to both erosion and abrasion.

#### In situ phase – enamel pellicle formation and modulation

Two adult female volunteers (23 and 24 years old) participated in the study after being screened for the following inclusion criteria: normal salivary parameters (stimulated salivary flow >1 ml/min; unstimulated salivary flow >0.1 ml/min; neutral pH levels, from 7.0-7.5) and good oral health (absence of dental erosion, periodontitis, or untreated carious lesions). Exclusion criteria included the presence of systemic diseases, exposure to radiotherapy or chemotherapy, use of medications affecting salivary characteristics (such as antidepressants, narcotics, diuretics, or antihistamines), gastroesophageal reflux or frequent regurgitation or vomiting, smoking, pregnancy or breastfeeding, participation in sports involving swimming pools (exposure to low pH treated water), and application of topical fluoride in the previous two months. Intraoral palatal appliances were fabricated from acrylic resin and had six sites (measuring 6x6x3mm^3^) for attaching six enamel blocks.

The enamel pellicle was formed in two different time periods, morning and afternoon. The appliance was placed in the oral cavity one hour after subjects completed their oral hygiene routine and left in place for 30 minutes (from 8:00 AM to 8:30 AM and from 1:00 PM to 1:30 PM).^34^ The appliance was then removed and the blocks were treated. Vitamin E and palm oil were used in their original formulations. A 2% concentration solution of proanthocyanidin was prepared. Because polyphenols, such as proanthocyanidin, have the ability to precipitate proteins from the pellicle, after the blocks were treated, the intraoral appliance was reinserted into the oral cavity and left in place for another hour.^35^

#### *In vitro* phase – treatment

The treatments were administered prior to the first and third erosive+abrasive cycles by applying five drops of the substances. These substances were left in contact with the enamel surface for 30 seconds. In cases in which test agents were combined, they were applied sequentially without rinsing. Treatment was performed on the enamel blocks while volunteers were wearing the appliance to prevent any damage to the previously formed AEP. After treatment, the palatal appliance was tilted at a 90-degree angle to facilitate drainage of excess solution. It was then reinserted into the oral cavity for another hour to facilitate further formation of enamel pellicle.^34^

#### *In vitro* phase – erosive and abrasive cycles

The blocks were subjected to four (4) erosive cycles daily for five (5) days by immersing the palatal intraoral device in 30 mL of 0.5% citric acid pH 2.5 for 90 seconds, with agitation at a speed of 45 rpm and room temperature of 25°C.

Half of the samples (42) were subjected to two abrasive challenges after the first and third erosive cycles. The blocks designated for erosion+abrasion were removed from the intraoral appliance after the erosive cycle and mounted on an acrylic disc. Abrasive cycling was performed in the Biopdi brushing machine (Dental Biopdi, São Carlos, Brazil) with 42 toothbrushes (Colgate Twister^®^, COLGATEPALMOLIVE INDUSTRIAL LTDA / São Bernardo do Campo / Brazil) that had been prepared in advance. The toothbrushes were held parallel to the tooth surface and the slurry solution was then automatically dispensed onto the specimen (~3 mL). The slurry solution was a dilution of fluoridated toothpaste (Colgate triple action^®^, COLGATE-PALMOLIVE INDUSTRIAL LTDA / São Bernardo do Campo / Brazil) in distilled water at a weight-to-volume ratio of 1:3, following the specifications of ISO 14569-1 (1999).^36^ Each abrasive challenge was performed for 15 s, with 45 back-and-forth movements (~3 back-and-forth movements per second),^37^ by reciprocal linear movement of the brushes and with the application of 150 g of weight on dental specimens, at a temperature of 37.5°C.^38,39^ When the brushing machine stopped, the specimens were removed, washed with deionized water for 5 seconds, and reattached to the device. They were then immersed in artificial saliva (0.33 g KH2PO4, 0.34 g Na2HPO4, 1.27 g KCl, 0.16 g NaSCN, 0.58 g NaCl, 0.17 g CaCl2, 0.16 g NH4Cl, 0.2g urea, 0.03 g glucose, 0.002 g ascorbic acid, pH 7 [Klimek, et al.^40^(1982)] modified, without mucin) for 2 hours until the next cycle. At the end of each cycle day, the specimens were placed in artificial saliva overnight (for 14 hours) at 37°C.

## Final profilometric analysis

After completing the erosive/abrasive cycles, the nail polish was removed and profilometric analysis was conducted using the same initial measurement parameters. The enamel samples were carefully positioned on the profilometer table, replicating their initial placement to ensure precise alignment of the initial (baseline) and final profiles. Subsequently, the profiles were superimposed and analyzed using a specific software program (MarSurf XCR 20, Göttingen, Germany). To quantify enamel loss, the vertical difference (mean surface depth) between the baseline and final surface profiles was measured and reported as the mean of five graphs.

## Statistical analysis

Statistical analysis was performed using STATISTICA data analysis software system version 11.0 (Stat Soft Inc.). The data showed normal distribution (Shapiro-Wilk test) and homogeneity, as confirmed by the Levene test. Two-way ANOVA with treatment and condition (E or EA) was then performed, followed by Tukey’s post hoc test. The significance level was set at 5%.

## Results

**Figure 2:**
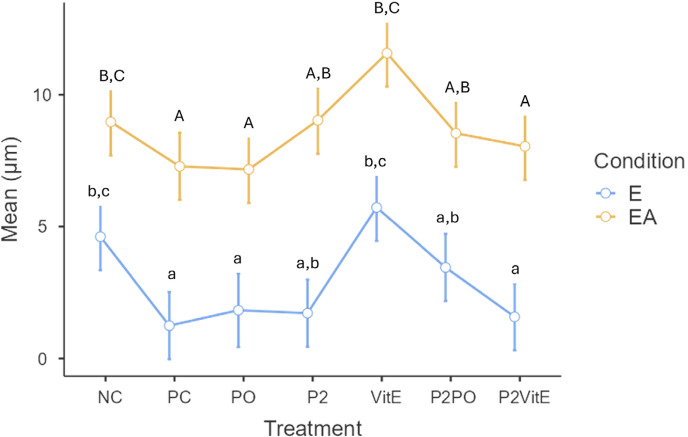
Graph showing the mean values of enamel wear (μm) and standard deviation. Different lowercase letters indicate statistical differences between treatments in the erosion (E) group (two-way ANOVA and Tukey test). Different uppercase letters indicate statistical differences between treatments in the erosion+abrasion (EA) group (two-way ANOVA and Tukey's test). Treatment (p<0.001), condition (p<0.001), and significant interaction (p=0.346).

Figure 2 shows the results. A significant difference was observed between treatments (p=0.001) and between conditions (p=0.0001), and there was no significant interaction between treatment types and conditions (p=0.346). The treatment groups PO, P2, P2VitE, and P2PO showed statistically similar enamel wear rates to the positive control group (PC), while only the PO and P2VitE groups differed from the negative control group (NC). Isolated vitamin E (VitE) was similar to the negative control group (NC) and showed greater enamel wear compared with the other groups. This pattern was maintained under erosion+abrasion; however, all treatment groups showed increased wear compared with those subjected to erosion only.

## Discussion

This study aimed to assess the effect of the combination of proanthocyanidin and vitamin E or palm oil on enamel against erosive and erosive+abrasive challenges *in vitro* using an *in situ* model of enamel pellicle formation. The combination of proanthocyanidin and vitamin E (P2PO) and isolated palm oil (PO) showed enamel wear similar to that of SnCl2/NaF/AmF-containing solution (Elmex^®^ Erosion Protection Dental Rinse) (PC), the positive control group. The combination of proanthocyanidin and palm oil (P2PO) was statistically similar to the positive and negative control groups. Meanwhile, vitamin E alone (VitE) was similar to the negative control group and showed the greatest wear among the treatments evaluated. The results also revealed that all study groups showed greater wear when subjected to the erosion+abrasion model. This increase in wear typically occurs when mechanical forces are applied to an enamel surface that has been softened by erosion, making it more susceptible to removal.^1,2,3,41^ Since the application of isolated and associated agents, except for isolated vitamin E, showed a potential protective effect against initial erosion and erosive tooth wear, both null hypotheses were rejected.

The favorable results observed with the combination of proanthocyanidin and vitamin E may be due to potential interactions between these components, such as the interaction between polyphenols and lipids.^42^ Therefore, we hypothesize that proanthocyanidin may have the ability to bind to lipids present in oily vitamin E and AEP, thereby contributing to the retardation of basal layer disintegration under erosive and abrasive challenges.

The ability of proanthocyanidin and vitamin E to interact with specific proteins within the AEP may also play an important role in enhancing the protection of AEP against erosive and abrasive challenges. On the one hand, proanthocyanidin has the ability to bind to serum albumin.^20,43^ Conversely, a potential mechanism attributed to the protective effect of vitamin E against enamel erosion involves the ability of this vitamin to interact with Afamin.^14,44,45^ Afamin is an alternative name for alpha-albumin, a specific protein within the serum albumin family^46^ that is frequently found in AEP.^47-51^ A previous study examined the interaction of proanthocyanidins and α-tocopherol (the most biologically active form of vitamin E) with human serum albumin (HSA) and found that these agents bind to different sites within this protein. Proanthocyanidin was found to bind to site I of HSA, while α-tocopherol was found to bind to site II.^38^ This suggests that there is no competition between these substances when administered concurrently, as both are capable of interacting with HSA.^43^ Therefore, proanthocyanidin and α-tocopherol may collectively induce balanced conformational and microenvironmental alterations in HSA.^43^ In light of this information, we postulate that there may be an interaction of proanthocyanidin and vitamin E with specific AEP proteins. However, additional investigations are needed to substantiate this hypothesis, such as studies performing a proteomic analysis of AEP modified with both vitamin E and proanthocyanidin. This analysis will serve to elucidate which proteins bind to these agents, thereby advancing our understanding.

In this study, isolated vitamin E (VitE) did not provide protection against enamel erosion and erosion+abrasion, in contrast to previous studies that assessed the effect of vitamin E on enamel erosion *in vitro*.^14,15^ This variance in results may be due to methodological differences between studies. In our study, specimens were treated with vitamin E twice a day, with applications occurring after 30 minutes of enamel pellicle formation^34,50^ and another hour of AEP modification with the use of the appliance.^35,50^ In addition, four erosive cycles of 90 seconds each were administered to all specimens, along with two abrasive cycles of 15 seconds each (applied to half of the specimens) daily for five consecutive days, resulting in a cumulative exposure of 30 minutes to erosion and of 2.5 minutes to abrasion. In Rios, et al.^14^(2021), enamel pellicle formation was conducted *in situ* using the appliance over a two-hour period. The specimens were then treated and immediately subjected to a single erosive cycle of 30 seconds. In Oliveira, et al.^15^(2023), treatment with artificial saliva before and after 1 hour of enamel pellicle formation proved effective in protecting against the proposed erosive challenge (1 minute, once a day, for 3 days). Therefore, it is plausible to hypothesize that vitamin E alone may not guarantee efficacy in severe challenges. Conversely, the enamel pellicle formation model proposed in this study may have limited its efficacy, as vitamin E may have been susceptible to rapid degradation after 1 hour of appliance reinsertion in the oral cavity.

The protective effect of proanthocyanidin has been attributed to the maturation and/or thickening of AEP, facilitated by the formation of proanthocyanidin-protein aggregates. Proanthocyanidin has the ability to induce precipitation and aggregation of salivary proteins,^21^ including proline-rich proteins (PRPs) and histatins.^20^ PRPs have the ability to form robust hydrogen bonds with proanthocyanidin.^13,20^ In parallel, histatins are able to form insoluble complexes with proanthocyanidin, and these complexes remain stable even under conditions mimicking the digestive tract with an acidic content (0.01 M HCl, pH 2.0).^13,51^ While Boteon, et al.^13^(2020) observed protective effects of proanthocyanidin against enamel erosion, in this study, the action of this compound was intermediate, that is, its behavior was similar to that of the positive and negative control groups. This discrepancy may be due to the increased severity of the erosive challenges. Boteon, et al.^13^(2020) evaluated the protective capacity of proanthocyanidin in a single short erosive challenge (30 s) by measuring the percentage of hardness loss. Therefore, we hypothesize that isolated proanthocyanidin may not fully manifest its protective potential against erosion and abrasion when exposed to prolonged erosive and erosive+abrasive challenges.

The Elmex Erosion Protection^®^ mouthwash, which contains stannous chloride, amine fluoride, and sodium fluoride, has been considered to be the gold standard product for the management of dental erosion, as indicated by previous research.^11,52,53^ Therefore, it was selected as the positive control group for this study. The effect of stannous fluoride on ETW control is attributed to its ability to incorporate fluoride and tin ions and modulate the acquired enamel pellicle (AEP) .^50,54,55^ Fluoride-modified AEP provides enhanced protection against demineralization and can even be incorporated into eroded enamel.^50,56^ Furthermore, stannous ions have the ability to interact with albumin, resulting in increased electron density in the basal layer of AEP, which can remain relatively unaffected following erosive challenges.^50,55^ In this study, the Elmex^®^ Erosion Protection (PC) mouthwash showed the lowest enamel wear, along with palm oil (PO) and the combination of proanthocyanidin with vitamin E (P2VitE).

The protective effect of palm oil against erosion and abrasion has been demonstrated in previous studies.^11,12,14^ Therefore, its satisfactory effect in this study was expected. It has been suggested that its protective effect may be due to the potential of palm oil to permeate the lipids within the basal layer of AEP, thereby retarding their disintegration.^11-12^ This phenomenon was primarily proposed due to the presence of tocotrienols, which are abundant in palm oil and have the ability to permeate cell membrane lipid layers due to the antioxidant properties of the oil.^57,58^

In this study, the combination of proanthocyanidin with vitamin E (P2VitE) yielded promising results. Although the erosive and erosive+abrasive cycles were performed *in vitro*, enamel pellicle was formed *in situ*, which is a positive aspect of this study. However, it would be interesting to evaluate these agents in a completely *in situ* study, simulating the biological conditions of the oral cavity, especially in relation to the constant renewal of salivary flow. In addition, the alteration of the AEP during its formation in our study may have affected the protective effect of the agents, especially vitamin E. Furthermore, exploring different models of enamel pellicle formation could help determine the optimal timing of treatment, in conjunction with proteomic analysis to elucidate the mechanism of interaction between the studied agents and AEP.

## Conclusion

In this study model, the combination of proanthocyanidin with vitamin E and isolated palm oil showed a remarkable ability to protect enamel against *in vitro* erosive and erosive+abrasive challenges. Notably, proanthocyanidin alone and in combination with palm oil showed intermediate protective effects, while isolated vitamin E did not show a protective effect. These findings elucidate potential methods for further exploration in the development of preventive strategies against erosive tooth wear.
